# Scientific evidence of sustainable plant disease protection strategies for oilseed rape (*Brassica napus*) in Sweden: a systematic map

**DOI:** 10.1186/s13750-022-00277-9

**Published:** 2022-06-21

**Authors:** Ann-Charlotte Wallenhammar, Elisa Vilvert, Sanna Bergqvist, Åke Olson, Anna Berlin

**Affiliations:** 1 Rural Economy and Agricultural Society Konsult AB, Gamla vägen 5G, 702 27 Örebro, Sweden; 2 Department of Forest Mycology and Plant Pathology, Swedish University of Agricultural Sciences, Box 7026, 750 07 Uppsala, Sweden

**Keywords:** *Brassica napus*, Rapeseed, Canola, Plant pathology

## Abstract

**Background:**

Oilseed rape (OSR; *Brassica napus* L.) is a highly valued crop for food, feed and industrial use. It is primarily grown in temperate climates, and over recent decades, its area of production and profitability have increased. Concurrently, several diseases negatively impact OSR production. Diseases caused by soil-borne pathogens, pose a risk of substantial yield loss since crop rotation schemes have become narrow as the time lapse between OSR crops in a field has been shortened. The aims of this paper were to provide an overview of plant protection measures available for OSR production and to identify knowledge gaps and areas where more research is needed.

**Methods:**

This systematic map builds on a previously published protocol and follows the ROSES reporting standard. The search strategy was developed in collaboration with stakeholders and designed to cover available scientific evidence for OSR disease management in climate zones relevant for Scandinavian crop production (Dfc, Dfb, Cfb and Cfa in the Köppen-Geiger climate classification). Five scientific databases were used to identify peer-reviewed literature, complemented by additional searches performed in grey literature. Articles were screened at three stages: the title, abstract and full text. The eligible publications included studies of OSR crops, and all measures to control crop disease in agricultural fields were considered eligible interventions. The comparator was intervention and no intervention, and the yield per unit area, disease suppression or an increase in crop quality were determined to be outcomes of interventions. A basic assessment of the experimental design of each study was performed to assess its eligibility. All articles were coded based on the following categories: the location and climate zone, disease, pathogen, intervention and management method, outcome and study design. Articles not reporting original data but judged to be relevant (i.e., review papers, books and notes of registration of cultivars) were saved in a separate category called “books, reviews and reports”.

**Review findings:**

A total of 4633 articles were collected through systematic searches. After duplicates were removed, 3513 articles were included in the screening process. After screening at the title and abstract levels, 897 articles were evaluated at the full text level, and 118 articles comprised the studies that met the eligibility criteria of the systematic map. The country (Canada) and region (Europe) with the largest OSR crop production areas also contributed the highest number of articles. In total, 17 different diseases were reported, with black leg (syn. Phoma stem canker) being the most studied disease. Nineteen different intervention methods or management types were examined. Cultivar resistance and pesticide application were the most studied control measures.

**Conclusion:**

We report scientific studies on plant disease protection measures for OSR based on field trials where the results are intended to be directly implemented in crop production management. The map clearly provides an overview of research progress throughout the time period chosen, and it identifies knowledge gaps regarding important diseases where only a few studies have been published, for example, diseases caused by viruses.

**Supplementary Information:**

The online version contains supplementary material available at 10.1186/s13750-022-00277-9.

## Background

With an increasing global population, there is an expected increase in the demand for vegetable oils. Oilseed rape (OSR; *Brassica napus* L.) is the third largest source of plant-derived oil in the world, with a global production of nearly 27.3 million metric tons annually [1]. The oil is highly valued because of its nutritional composition [2] and is considered one of the healthiest vegetable oils for human consumption. The residue after oil extraction, rapeseed meal, constitutes a valuable source of animal feed due to its high protein content. The production of OSR is steadily increasing, and this trend is predicted to continue, particularly since a growing consumer health consciousness is causing a shift towards the use of vegetable oils [3]. Worldwide, approximately 60%, 38%, and 3% of rapeseed oil is used for food, industrial uses, and feed, respectively. These proportions, however, vary significantly between regions [4].

Since prehistoric times, many *Brassica* species have been cultivated for their edible roots, stems, leaves, buds, flowers and seeds. Although OSR cultivation started in the thirteenth century in Europe, industrial use was not widespread until its superior qualities as a lubricant oil were realized in the 1930s [5]. Its use as an edible oil is even more recent [5], as the nutritional properties of past OSR cultivars were modest, with the high content of erucic acid and glucosinolates giving the oil an unpleasant and bitter taste. Cultivars with low glucosinolate and erucic acid content were developed via conventional breeding and were initially produced in Canada under the trade name Canola, defined as “an oil that must contain less than two percent erucic acid, and less than 30 micromoles of glucosinolates per gram of air-dried oil-free meal”. Canola has since become the common term for these ‘double-low’ cultivars in North America [4].

**Fig. 1:**
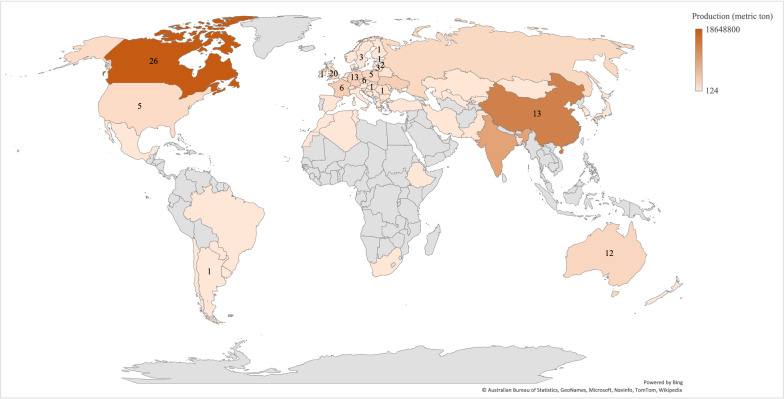
Geographical location of the articles included in the systematic map and global oilseed rape production. The countries with articles included in the systematic map are indicated by numbers that refer to the number of articles conducted by each country. The countries in colour indicate the countries with oilseed rape production in 2019 [1]

Oilseed rape is primarily grown in temperate environments (Fig. 1). The production of rapeseed has increased over recent decades throughout the main production regions: Canada, Europe, China, India and Australia [1]. *Brassica napus* and *B. rapa* are the two OSR species grown in Europe and Canada. On the global scale, there is no discrimination in the harvested seed market between *B. napus* and *B. rapa.* There are spring- and autumn-sown varieties of both species, providing growers with a choice of four crop types: spring OSR, spring oilseed turnip rape (OTR; *Brassica rapa*), winter OSR and winter OTR*.* In most situations, *B. napus* is higher yielding than *B. rapa*; however, *B. rapa* matures earlier [6]. Winter OSR (*B. napus* var. *biennis*) is grown in regions where the crop escapes from winter kill but where the regions are classified as Cfb and Dfb. In regions with harsh climatic conditions during winter, spring OSR and spring OTR are grown. In Sweden, in recent decades, the production of OTR has been reduced in favour of OSR, and OTR is currently grown on only two percent of the total rapeseed acreage [7].

Oilseed rape is a highly profitable crop when managed correctly, and the benefits of planting OSR before a cereal in crop rotation schemes are widely reported [8–10]. The effects include subsoil amelioration, which leads to increased nutrient and water uptake. In addition, as a pre-crop, OSR suppresses diseases in cereals [10]. OSR is frequently grown in short rotations; one out of every three years in arable rotations in Scotland and England and every second year are reported [11]. In western Canada, the production of canola crops has increased substantially, and thus, crop diversity has been reduced [12].

The introduction of high-yielding cultivars has increased the profitability of OSR [13]; however, several diseases and insect pests negatively influence production. In a global survey conducted in 2019 [13], the main biotic constraints were reported to be caused by soil-borne pathogens such as *Plasmodiophora brassicae* (clubroot) and *Verticillium longisporum* (Verticillium wilt) and by pathogens that affect the stem such as *Sclerotinia sclerotiorum* (Sclerotinia stem rot)*, Lepthospaheria maculans* and *L. biglobosa* (black leg or Phoma stem canker), *Alternaria* spp. (black spot, dark leaf and pod spot), *Pseudocercosporellae capsellae* (white leaf spot) and *Pyrenopezizia brassicae* (light leaf spot). Some of the diseases are reported only in certain regions, whereas others, such as Sclerotinia stem rot, are reported to cause important yield reductions in all major OSR growing regions in the world [13]. A high frequency of OSR in crop rotations increases the risk of propagating and spreading soil-borne diseases by movement of infested soil via equipment into new fields, such as clubroot disease, which has become a serious constraint on OSR production worldwide [14]. The increasing spread of clubroot in the major production regions of Canada, the UK, Germany, Poland, the Czech Republic, China and other countries is a consequence of the practice of narrow crop rotations due to the increasing demand for rapeseed oil [11, 15–21]. At present, approximately 100,000 hectares of winter OSR and 6000 hectares of spring OSR are grown in Sweden, and in recent years, there has been a shift to winter OSR in regions where spring OSR was previously grown. Consequently, new diseases associated with winter OSR, such as black leg, are emerging in these regions [22]. Sclerotinia stem rot and clubroot are important diseases in all regions of Sweden [23, 24], causing yield reductions if not managed properly.

The increased production of OSR due to higher demands for rapeseed products requires strategies that combine different means of control, including pathogen avoidance, pathogen exclusion, host plant protection and host resistance [25]. Knowledge of plant disease epidemiology is important for selecting the most effective management method or combination of methods for controlling crop diseases. Each pathogen has a unique, often complicated, life cycle, and growers need to make informed decisions to avoid disease outbreaks. Plant protection management is multifaceted, and a range of factors must be considered by growers, such as the choice of cultivars and agronomic practices. Often, the use of chemical interventions is the only direct measure of limiting the negative impact of a plant disease within a growing season. The EU directive on the sustainable use of pesticides (2009/128/EC) encourages integrated pest management (IPM). IPM calls for crop protection strategies that combine preventive measures such as disease outbreak prediction, a crop rotation where the target crop is grown several years apart, and cultivar selection in combination with direct measures such as pesticide use.

### Topic identification and stakeholder engagement

Measures for the disease control of crops in Sweden are often based on a combination of regional field trials, scientific evidence and practical experience. Comprehensive recommendations based on scientific results are needed to meet the challenges faced by crop producers. The aims of this study were to provide an overview of the available plant disease protection measures for OSR that are supported by scientifically based evidence and to pinpoint knowledge gaps and thus identify areas where more information is needed to advance plant protection in OSR production. When developing the primary question, the topic and relevance were actively discussed with researchers, a stakeholder representative from the Plant Protection Centers at the Swedish Board of Agriculture and farmers’ advisory service. In the development of the eligibility criteria, the group was also consulted.

## Objective of the review

OSR is an important crop for Swedish agriculture, and this map was developed to compile research literature of relevance for Swedish conditions. The objective was to identify the evidence base for plant disease protection measures and the strategies available for OSR production in Sweden. The systematic map describes the volume and main characteristics of field-based plant protection research based on systematic searches in scientific databases and grey literature searches. The overall aim was to provide an overview of scientifically supported plant disease protection measures for OSR, to identify knowledge gaps and areas where more research is needed and to provide a knowledge base for plant protection specialists as well as research councils and policy decision-makers when allocating resources for research. The map is based on the protocol previously published by Berlin et al. [26] and includes articles from the climatic zones relevant for Swedish OSR production. In Sweden, arable lands falls into three different climatic zones based on the Köppen-Geiger climate classification [27]. Dfc and Dfb are classified as cold temperate (D) and fully humid (f) climates and vary by a cool summer (c) and a warm summer (b), and Cfb is classified as a warm temperate (C) and fully humid climate (f) with a warm summer (b). The climate zone Cfa representing a warm temperate (C) and fully humid climate (f) with a hot summer (a) was also included since this type of climate is expected to occur in southern Sweden in the future because of climate change.

The primary question was: *What is the evidence base of plant disease protection measures and strategies available for oilseed rape in Sweden?*

The question has the following components:

### Population

Oilseed rape (*Brassica napus*) crops in climatic zones (Dfc, Dfb, Cfb and Cfa) relevant to Swedish OSR production.

### Intervention

Any measure for controlling crop diseases in agricultural fields, including both direct and indirect interventions.

### Comparator

No intervention (control) and between different interventions.

### Outcomes

The yield or outcome measured as the yield per unit area, disease suppression and/or an increase in crop quality.

## Methods

The systematic map was developed based on the protocol previously published by Berlin et al. [26]. The protocol follows the Collaboration for Environmental Evidence Guidelines and Standards for Evidence Synthesis in Environmental Management [28] and conforms to the ROSES reporting standard [29] (see Additional file 1). In brief, this section describes the design and details of the systematic map for OSR. All deviations from the protocol are described below.

### Deviations from the protocol

The methods in this map deviate from protocol [25] in the following ways:

- The protocol was designed for six crops; however, this map catalogues information about only OSR. The other crops listed in the protocol [30] will be considered for publication in separate systematic maps.
- To facilitate the Google Scholar search, Publish and Perish [31] software was used.
- Norwegian and Danish were included as relevant languages due to their relevance within the Nordic region and the knowledge of the languages in the expert group.
- The search terms for grey literature were adjusted based on the webpages of organizations, and the procedure for conducting the search is described in the methodology below.
- The list of papers, including those removed at the title, abstract and full text levels, is provided as an Excel file, as opposed to being included in the EndNote library file in the additional material.
- The intervention and management data coding was merged in the analysis of the results since several studies used more than one type of agricultural practice (described as management) as intervention methods for disease control.
- The publications included in the category “books, reviews and reports” were categorized into sub-categories as described below.

### Search for articles

An extensive search for literature was conducted in academic bibliographic databases and in relevant online sources for grey literature. The search was conducted in English and included the scientific name of the crop. The search string developed for the scientific databases was structured into four thematic blocks: crop, disease-causing organisms, plant disease terms and outcomes. The blocks were combined using the operator “AND” to obtain the final search string (Table 1).

**Table 1:** Search string with four thematic blocks combined using the operator “AND”

Thematic block	Search string
Crop	Oilseed rape OR “*Brassica napus*”
Disease-causing organisms	Fung* OR oomycete* OR nematod* OR bacter* OR virus* OR viral OR viroid* OR pathogen*
Plant disease terms	“Disease incidence” OR “disease severity” OR “plant protection” OR “control strateg*” OR “risk management” OR “biological control” OR “disease control” OR IPM OR “integrated pest management” OR “plant defen*” OR resistance OR “disease develop*”
Outcomes	“Plant health” OR yield* OR qualit* OR harvest OR produc* OR “pathogen reduction”

A shorter search string was created for AGRIS and Google Scholar to adapt to the requirements of these databases (Fung* OR oomycete* OR nematode *OR bacter* OR virus* OR viral* OR virioid* OR pathogen*) AND (Oilseed rape OR “*Brassica napus*”).

A time span restriction that included literature published during the past 40 years (1978–2018) was applied. For all searches, the following data from the search process were recorded: the date of the search, the database and platform name, the institutional subscription used to access the database, the search string, and the number of hits for each search (Additional file 2). The following academic bibliographic databases were used to search for articles:

- Web of Science Core Collection (http://webofknowledge.com/WOS).
- Biosis Citation Index (http://webofknowledge.com/BCI).
- CABI: CAB Abstracts and Global Health (http://webofknowledge.com/CABI).
- Scopus (https://www.scopus.com/).
- AGRIS (http://agris.fao.org/).

Web of Science Core Collection, Biosis Citation Index and CABI: CAB Abstracts and Global Health was accessed through the Swedish University of Agricultural Sciences (SLU) subscription to Web of Science (v.5.30). Scopus and AGRIS were accessed directly through their websites. A search in Google Scholar using Publish or Perish software [31] was performed to extract the first 1000 search results, and these results were merged with the results from the five academic bibliographic databases.

The grey literature search was performed to cover different types of sources. First, three databases for pre-print archives were used to identify pre-published research studies: bioRxiv (http://www.biorxiv.org), PeerJ (http://www.peerj.org) and arXiv (http://www.arxiv.org). Then, searches on 21 webpages of relevant organizations listed in Additional file 2 were performed. For the search in organizational webpages, a more limited search was conducted using just the crop name in English and in Latin, i.e., “Oilseed rape” and “*Brassica napus*”. For Swedish, Norwegian and Danish webpages, searches were also performed using the crop name in the respective languages, whereas for Finnish webpages, searches were performed using both the Swedish and English versions of the webpages.

### Article screening and study eligibility criteria

The results from the searches were imported and combined into one EndNote X9 library file. With the exception of the search in the databases for preprint archives and on the webpages of organizations, for which the screening process was conducted directly on the respective webpages, only the articles selected at the full text level were included in the EndNote X9 database. First, duplicates were removed and recorded in a separate folder, and then all results written in an ineligible language were removed and recorded.

#### Eligibility criteria

The eligibility criteria were defined prior to screening to ensure that only articles eligible based on the objective were included in the systematic map. All retrieved articles were assessed for relevance using the eligibility criteria described in Table 2.

**Table 2:** Description of the eligibility criteria: population, climate, language, intervention, outcome and study type(s) in the screening process

Eligible criteria	Included	Excluded
Population	Studies on oilseed rape crops (*Brassica napus*) conducted in the field	Studies not including *B. napus* or including oilseed rape (*B. napus*) but performed in laboratories or greenhouses, as pot experiments or based on GMO cultivars or lineages
Climate	This inclusion criterion was based on the Köppen-Geiger climate classification [27]. Studies from temperate regions in zones Cfb and Dfb, corresponding to the main agricultural areas of southern Sweden, and Dfc, corresponding to northern Sweden. Studies from regions in the climate zone Cfa since this climate is expected to prevail in Sweden due to climate change	Studies conducted in a geographical region with no relevant climate based on the Köppen-Geiger climate classification zones
Language	Full text in English, Swedish, Norwegian or Danish	Any study not available with its full text in one of the relevant languages
Intervention^a^	Any disease management intervention, independently or in combination, including but not limited to crop rotation, resistant cultivars, cultivar mixtures, ploughs, no-tillage, biological control, and biofungicide and pesticide applications	Studies that applied pesticide active substances not allowed for use against plant diseases in the EU. For this purpose, a list of active substances allowed for use in the EU was retrieved from the European Commission database (https://ec.europa.eu/food/plant/pesticides) at the start of the article screening process (Additional file 3)
Outcome	Any type of effect of disease control interventions measured by productivity in terms of total harvest, the yield per unit area or relevant crop quality measures, e.g., plant health status or reduced disease symptoms. Disease reduction was included as a proxy for a potential yield increase or an increase in crop quality	
Study design	Use of any relevant experimental study design including but not limited to before and after (BA) studies, randomized control trials (RCTs) randomized split block trials (RSBTs) and exposure versus no exposures/control impact (CI)	Articles and reports not including original data and/or with no relevant study design or experimental data not statistically evaluated

^a^The intervention criteria are described in detail in Table 3

When there was doubt about the relevance of an article at any of the screening stages, the article was included. Articles with no abstract assigned by the scientific database to the EndNote library and not found through online searches were excluded. Articles that were not accessible as full text online (through the SLU subscription or as open access) or available as printed versions through the SLU library were also excluded (see reasons for exclusion in Additional file 4).

#### Screening process

Article screening was conducted at three levels: (1) the title, (2) abstract and (3) full text. At each level, the articles were assessed following the eligibility criteria (Table 2). To ensure consistency between the reviewers in the screening process [26], a set of 200 articles was screened independently by all authors to validate the consistency of the eligibility decisions against the eligibility criteria at the title, abstract and full text levels. The screening process was validated by a kappa test [32] at all three levels, and the kappa test showed fair agreement (0.27 and 0.30, respectively) at the title and abstract levels. At the full text level, initially, the kappa test also showed fair agreement (k = 0.32). After discussions of the disagreements and clarification of the eligible criteria at all levels, however, the reviewers re-analysed the articles on which they had first disagreed and re-evaluated them. Then, a kappa score of 0.62 was obtained, which is in accordance with the expected score defined in the systematic map protocol, showing substantial agreement. The few remaining disagreements were discussed and re-evaluated jointly by all reviewers until a common agreement was reached.

The complete screening process for the articles obtained from the academic bibliographic databases was conducted by the same two independent reviewers at all three levels. At the end of the screening process, the consistency of the eligibility decisions on all included articles between the two reviewers was compared. The results were validated by a kappa test at the full text level [32], and a kappa score of 0.61 (substantial agreement) was obtained. All remaining discrepancies at the full text level were discussed and re-evaluated until a common agreement was reached.

All articles excluded after screening at the title and abstract levels were recorded in a separate list (Additional file 4). Articles excluded at the full text level were recorded in a separate list and assigned a reason for exclusion (Additional file 4).

Eligible review articles, books, reports (with no relevant study design or experimental data not statistically evaluated), conference papers and notes of registration of cultivars were recorded in a separate file named “books, reviews and reports” (Additional file 4) and were not included in the systematic map. However, the reference lists of these articles were screened, and the research articles not identified in the database searches and fulfilling the eligibility criteria were added to the systematic map database.

The screening process for grey literature was conducted by just one reviewer, and a second reviewer checked and validated the articles selected at the full text level. Reviewers who were authors of eligible articles were not included in the decision connected to the inclusion and critical appraisal of their articles.

### Study validity assessment

The identification and assessment of the experimental design were performed when evaluating the relevance of a study and if it was eligible to be included in the map following the eligibility criteria described in Table 2. No further study validity assessment was performed because the intention of this map is to provide an overview of the available literature about disease control methods in OSR production.

### Data coding strategy

Standardized descriptive data from all articles meeting the eligibility criteria were exported from EndNote to an Excel spreadsheet, forming the systematic map database (Additional file 5). The coding of the intervention and management methods was carefully discussed and defined (Table 3). The following data variables of interest were extracted from the articles included in the systematic map: (1) bibliographic information; (2) climatic zones (s); (3) the location(s) of the study; (4) disease name(s); (5) pathogen type(s); (6) disease-causing organisms; (7) the intervention and management method; (8) the diseased part and plant development stage; and (9) the approach for measuring the outcome (for details on the coding strategy, see Additional file 5).

**Table 3:** Description of the disease control intervention and management strategies applied in the studies included in the systematic map

Intervention/management	Description
Biological control	Studies including any approved biological control agent (for use in the EU) for disease control. Includes tests with different spraying rates and times and comparisons between different biological control agents
Crop rotation	Use of crop rotation for disease control management
Cultivar resistance	Assessment of different oilseed rape cultivars for resistance to diseases
Defence elicitor	Use of any kind of plant defence elicitors that can induce host defence responses against a plant disease (β-aminobutyric acid, cis-jasmone, chitinosan, acibenzolar-S-methyl, etc.)
Defoliation	Use of mechanical defoliation of infected leaves as a disease control management to reduce the disease inoculum on plants and the potential for disease dispersal
Degree of soil infestation	Test of different levels of disease inoculum in the soil to evaluate the effects on disease development
Distance to inoculum	Analysis of disease development dependent on the distance between infested fields (inoculum source) and fields with no disease to evaluate the wind dispersal of inoculum between fields
Harvest techniques	Use of different harvest techniques (swathing versus straight combining) at different harvest dates to assess seed infection
Pesticides	Use of approved pesticides (for use in the EU) for disease control, including fungicides, herbicides and insecticides. Includes tests with different spraying rates and times, the type of adjuvant, the nozzle type used for applying the pesticide, and comparisons between different pesticides
Plant density	Use of different plant densities to assess the disease incidence in the field
Seeding depth	Use of different seed depths to assess the effect on crop performance in diseased plants
Seed size	Use of different seed sizes to assess the effect on crop performance in diseased plants
Soil amendments	Studies including any kind of fertilizers and soil amendments for disease control (nitrogen, boron, calcium, wood ash, etc.). Includes tests with different application rates and times of application
Stubble management	Any type of crop residue management used to reduce disease incidence

For articles that included more than one disease, all diseases were noted as were the other data variables. Articles that included other crops in addition to OSR were included in the systematic map, but only information regarding OSR was reported. The coding process was conducted by one reviewer and checked by a second reviewer, and any ambiguities were discussed and resolved.

The articles included in the folder “books, reviews and reports” were categorized into five different groups: review articles in scientific journals, books or book chapters, conference contributions, reports (with no relevant study design or not statistically evaluated) and notes of registration of cultivars.

### Data mapping method

All articles included and their metadata were recorded in a searchable Excel database available in Additional file 5. A few articles included more than one study, and here, the number of articles and not individual studies are registered. Some articles included studies with experiments in multiple countries or climate zones. In other articles, multiple diseases and management methods were studied.

A map of the OSR production in different countries and the number of articles from different locations was produced using the map chart featured in Excel (Fig. 1). Figures and tables for summarizing the interventions and management categories identified were produced to visualize the results. The number of publications per year, the type of pathogen and name of disease and interventions are visualized in figures, while the articles on each climatic zone, the diseases per country, and biocontrol and fungicide control measures are presented in tables.

## Review findings

### Search results

**Fig. 2:**
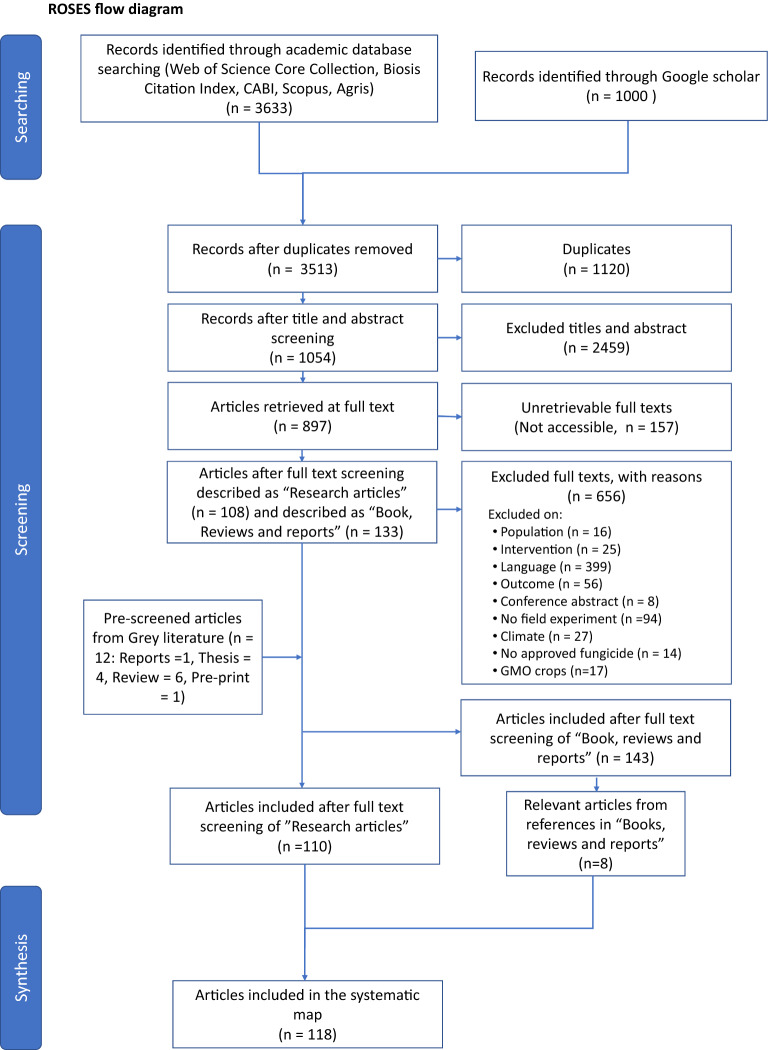
ROSES flow diagram illustrating the literature search and screening process

In total, 3633 scientific records were downloaded from the five academic databases, and the top 1000 records were downloaded from Google Scholar, resulting in a total of 4633 results (Fig. 2). After duplicates were removed, 3513 articles were included for the screening process.

In total, 2459 articles were removed at the title and abstract levels, 656 records were removed at the full text level, and 157 records were not accessible at the full text level. The reasons for exclusion at the full text level were ineligible language (n = 399), experiments not conducted in the field (n = 94), no eligible outcome (n = 56), no eligible climate (n = 27), no eligible intervention (n = 25), the use of GMO crops (n = 17), studies conducted with species other than *Brassica napus* (n = 16), the use of unapproved fungicides (n = 14) and conference abstracts without the possibility of evaluating the experimental design (n = 8). From the scientific database searches, 108 articles met the eligibility criteria and were included in the systematic map. In addition, 133 eligible articles were categorized as informative articles and saved under “books, reviews and reports”.

The grey literature pre-screening on the organizational websites resulted in 12 eligible articles, of which two articles were eligible for the systematic map and 10 articles were included in the group “books, reviews and reports” (n = 143). The reference lists of the articles included in “books, reviews and reports” were screened, and eight additional articles not found in the database searches were identified as eligible and included in the systematic map database.

Ultimately, 118 articles were eligible in the systematic map: 114 original scientific journal articles, two master’s theses, one doctoral thesis and one article from a pre-print archive. The articles were published by 48 different journals, affiliated with three different universities and uploaded onto one pre-print archive website. All eligible articles were written in English.

### Temporal and geographical distribution of research

**Fig. 3:**
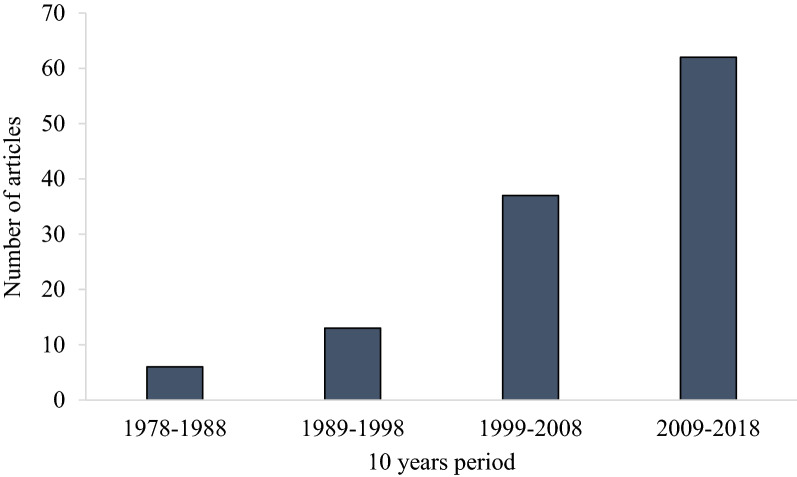
Number of articles published in the ten-year periods (1978–2018) included in the systematic map

The number of articles published in ten-year periods throughout the study period 1978–2018 is displayed in Fig. 3. The number of eligible articles increased over time, and 54% of the articles were published during the last decade, which correlates with the expansion in production during the time frame of the searches [1]. It should be noted that in one article, field trials were conducted in three different countries within the same climatic zone. Similarly, for eight articles, experiments were conducted in two different climatic zones (Table 4).

**Table 4:** Number of articles in relevant climatic zones per country included in the systematic map

Location of study	Cfb	Dfb	Cfa	Dfc
Canada		24		2
UK	20			
Australia	7		9	
Germany	13	1		
China			13	
Czech Republic	5	2		
France	6			
Poland	5	1		
USA		5		
Lithuania		3		
Sweden	1	2		
Finland		1		1
Latvia		2		
Estonia		1		
Ireland	1			
Argentina			1	
Romania		1		
Hungary	1			
Total	59	43	23	3

The geographical distribution of the included articles and the number of articles per country are presented in the map (Fig. 1). Most of the articles were conducted in the Northern Hemisphere, with Canada contributing the highest number of articles (n = 26), followed by the UK (n = 20), China (n = 13), Germany (n = 13) and Australia (n = 12) (Fig. 1). By continent, Europe contributed the highest number of articles (n = 63).

**Fig. 4:**
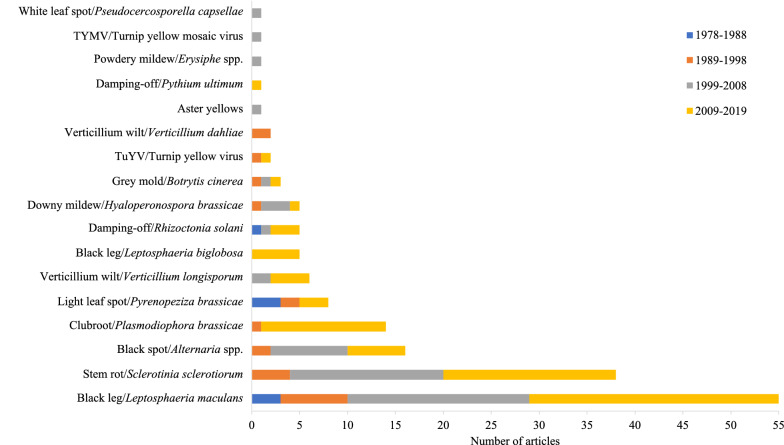
Number of articles included in the systematic map per disease analysed and categorized in ten-year publication periods (1978–1988, 1989–1998, 1999–2008 and 2009–2019)

In the first ten years within the search period of this map (1978–1988), only articles studying three diseases were included: one study of damping-off and three studies of light leaf spot and black leg (Phoma stem canker) (Fig. 4). During the following ten-year period (1989–1998), articles including studies of nine pathogens were included: Verticillium wilt, turnip yellow virus (TuYV), grey mould, downy mildew, light leaf spot, clubroot, black spot, Sclerotinia stem rot and black leg. In the next 10-year period (1999–2008), eleven diseases were included in articles eligible for the systematic map. A few studies were performed on aster yellows, powdery mildew, white leaf spot, turnip yellow mosaic virus (TYMW), grey mould, downy mildew and damping-off and Verticillium wilt, while most of the studies that were performed concerned black spot, Sclerotinia stem rot and black leg. During the last 10-year period (2009–2018), articles including studies on twelve diseases were included in the systematic map, and most of the studies concerned black leg (Phoma stem canker) caused by *L. maculans*. During this period, studies on black leg caused by *L. biglobosa* appeared for the first time. The number of studies of Sclerotinia stem rot performed was on the same level as in the previous period, whereas the number of studies on clubroot showed a large increase, with 13 studies included in the systematic map, reflecting the consequences of the outbreak of clubroot disease in canola crops in the state of Alberta, Canada, in 2003 [15].

Concerning the climatic zones, the majority of studies were conducted in regions situated in the Cfb (n = 59) and Dfb (n = 43) climatic zones (Table 4), corresponding to the temperate climate prevailing in large parts of Canada and Europe.

### Pathogen type and diseases studied

A compilation of the disease-causing organisms analysed in the articles showed that in 82% of the articles included in the systematic map fungal pathogens were the cause of disease, while 11% of the articles included diseases caused by a protist, 4% of the articles concerned diseases caused by oomycetes, and 3% of the articles addressed diseases caused by viruses and virus-like organisms. It should be noted that six articles analysed experiments with two different pathogen types, and 23 articles analysed two or more diseases.

In total, 17 different diseases were reported in the articles included in the systematic map. Figure 4 provides an overview of the number of articles reporting different diseases by decade. Thirteen diseases were described by common names, of which black leg (called Phoma stem canker in articles from the UK) is caused by pathogen populations comprising two main species: *L. maculans* and *L. biglobosa*. These pathogens were the most studied, with 60 articles included in the systematic map, corresponding to 37% of the total number of articles (Fig. 4). Black leg is an economically important disease in Europe, North America and Australia [13], which is reflected in the number of articles studying this disease over time (Table 5), and the disease has received increasing attention from plant pathologists across the world. The *Lepthospheria* species complex was historically divided into two groups of isolates, A (highly virulent) corresponding to *L. maculans* and B (weakly virulent) corresponding to *L. biglobosa*. In recent years, *L. biglobosa* has also been considered to cause yield reductions [33].

**Table 5:** Number of articles included in the systematic map categorized per disease and the country in which the disease was studied

Disease	Black leg^a^	Stem rot	Black spot	Club root	Light leaf spot	Verticillium wilt	Damping-off	Downy mildew	Grey mould	TuYV	Aster yellows	Powdery mildew	TYMV	White leaf spot	Total
UK	14	3	2	1	8	1		1	1	1					32
Canada	8	6	3	9			4				1				31
Germany	8	2	1			5		1	1	1				1	20
Poland	7	2	4			1			1						15
China		12		1			1								14
Australia	11	2													13
Czech Republic	3	3		1									1		8
France	6														6
Latvia	2		2				1	1							6
USA		5													5
Hungary	1	1	1					1							4
Lithuania	1		2												3
Sweden		1		1		1									3
Romania								1				1			2
Argentina	1														1
Estonia			1												1
Finland				1											1
Ireland		1													1
Total	62	38	16	14	8	8	6	5	3	2	1	1	1	1	

^a^Phoma stem canker

Sclerotinia stem rot is caused by a pathogen with close to 400 host species among dicotyledonous plants, affecting the stem in OSR and leading to substantial yield reductions [34]. For thirty-eight articles included in the systematic map studies were conducted on this pathogen (Fig. 4). Research activities on Sclerotinia stem rot have increased over time, from four articles in the 1990s to 16 and 18 articles in the last two 10-year periods (Fig. 3). In the articles, most studies of stem rot were performed in China (n = 12), followed by Canada (n = 6), the USA (n = 5), the UK (n = 3) and the Czech Republic (n = 3) (Table 5).

For Verticillium wilt, two pathogens have been reported as the causative organism. In the studies performed on Verticillium wilt in the period 1989–1998, *Verticillium dahliae* was considered the causative pathogen. In the next 10-year period and thereafter, the causative pathogen was referred to as *V. longisporum* [35], as it was later reclassified as this species [36].

Black spot, caused by *Alternaria* spp., was the subject of 16 articles (Fig. 4), where four studies were performed in Poland, followed by three studies in Canada and two studies in the UK, Latvia and Lithuania (Table 5). For black spot, the number of articles reported (n = 8 in 1999–2008) decreased during the last ten-year period compared to the previous period (n = 6) (Fig. 4).

Clubroot, a disease caused by the protist *P. brassicae* producing long-lasting resting spores [37], was the subject of 14 articles, all of which were performed in Canada. Clubroot has become an important disease, reflected by an increase of 130% in the number of publications regarding this disease during the last 10 years.

For light leaf spot caused by *Pyrenopezizia brassice*, a disease mainly reported by researchers in the UK, eight articles were included. Eight articles were performed on Verticillium wilt (*Verticillium longisporum* and *V. dahlie*), five of which were carried out in Germany. Different pathogens, including *Rhizoctonia* spp. and *Pythiu*m spp., contributes to damping-off symptoms in seedlings, and altogether, six articles were included. For downy mildew, five articles were included; for grey mould, three articles; for TuYV, two articles were included respectively. For aster yellows, powdery mildew, TYMV and white leaf spot, one article reporting each disease was included in the systematic map. TuYV is probably the most important viral disease of OSR in the UK [38]. Virus symptoms, which are not readily recognizable, are usually not visible before stem extension and can easily be confused with other stress symptoms and nutritional deficiencies. The main virus vector is the peach-potato aphid, *Myzus persicae*, and climate change is expected to exacerbate the situation as the conditions for the survival and multiplication of the vector are likely to be enhanced [38]. Only two articles (one from the UK and one from Germany), however, were included in the systematic map.

### Intervention type

**Fig. 5:**
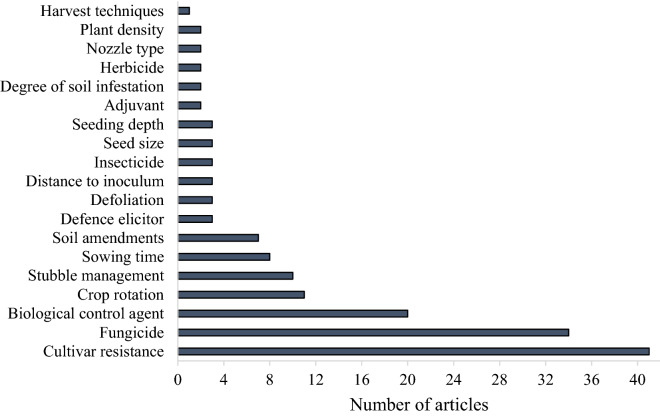
Number of articles included in the systematic map for the interventions type tested

The number of articles included in the systematic map related to the type of experimental intervention is presented in Fig. 5. Altogether, 19 different intervention methods or types of management to control diseases were eligible for inclusion in the systematic map. It should be noted that in 28 articles, experiments were conducted with more than one intervention type. The majority of the articles focused on ‘cultivar resistance’ (n = 41), followed by pesticide application (fungicide, insecticide and herbicide, n = 39). Interventions with ‘biological control agents’ (n = 20) were developed to control Sclerotinia stem rot (Table 6). Other types of interventions were mainly related to agricultural management practices, such as ‘crop rotation’ (n = 11), stubble management (n = 10), sowing time (n = 8) and soil amendments (n = 7).

**Table 6:** Number of articles included in the systematic map for each intervention and the targeted diseases

Intervention type	Black leg^a^	Stem rot	Black spot	Club root	Damping-off	Light leaf spot	Verticillium wilt	Downy mildew	Grey mould	TuYV	Aster yellows	Powdery mildew	TYMV	White leaf spot	Total
Fungicides	23	8	7	2		6	1	1	2						50
Cultivar resistance	26	7	3	6		1	3		1				1		48
Biological control agent	3	15	1	1	2		2	1							25
Crop rotation	8	3	2	2		1	2		1						19
Stubble management	8	1	1		1		1	2	1			1		1	17
Sowing time	3	2	1	1	3			1							11
Soil amendments	1	2	1	4							1				9
Defence elicitor	1					2									3
Defoliation	3														3
Distance to inoculum	2	1													3
Insecticide	1									2					3
Seed size		1			2										3
Seeding depth		1			2										3
Adjuvant	2														2
Degree of soil infestation				1			1								2
Herbicide	1					1									2
Nozzle type	2														2
Plant density		2													2
Harvest techniques			1												1

^a^Phoma stem canker

Host resistance developed in breeding programmes is an important method of controlling crop diseases and is considered one of the most economical and environmentally friendly methods for this purpose [39]. The two types of cultivar resistance used are quantitative resistance and qualitative resistance. Quantitative resistance, which is usually controlled by several genes, is often a ‘partial’ resistance that does not prevent pathogens from colonizing plants but decreases symptom severity and/or epidemic progression over time. By contrast, qualitative or ‘complete’ resistance is usually controlled by a single dominant resistance (*R*) gene and is often effective in preventing pathogens from colonizing plants. Following a gene-for-gene interaction, it is generally less durable since pathogen populations often rapidly evolve for virulence against the *R* genes [39]. For cultivar resistance, black leg is the most studied disease, as it was included in 26 articles (Table 6). The complexity of the disease, where the stem canker symptoms are initiated from lesions on the leaves of winter OSR in the previous autumn, has led to breeding activities based on qualitative resistance due to the incomplete nature of quantitative resistance [39]. For clubroot, control using resistant cultivars is the ultimate tool [37] for this protist [40] where no chemical interventions are available. Six articles that analysed the use of clubroot resistance were included. For stem rot, seven articles regarding cultivar resistance were included in the systematic map; for black spot and Verticillium wilt, three articles were included; and for light leaf spot, grey mould and TYMV, one article for each was included in the systematic map.

Fungicide was the most applied pesticide type and was used to control Phoma stem canker or black leg (n = 23), Sclerotinia stem rot (n = 8), black spot (n = 7) and light leaf spot (n = 6) (Table 6). The number of articles per type of pesticide active substance and the targeted disease are displayed in Table 7.

**Table 7:** Number of articles included in the systematic map per type of pesticide active substance and the targeted disease

Pesticide active substance	Black leg^a^	Stem rot	Light leaf spot	Black spot	Club root	Grey mould	TuYV	Verticillium wilt	Downy mildew	Total
Tebuconazole	5	4	3	3						15
Boscalid	3	5	1	2		1		1		13
Prochloraz	4	1	3	3		1			1	13
Azoxystrobin	4	4	1	1		1		1		12
Metconazole	6	1	2	2						11
Prothioconazole	3	4	3							10
Dimoxystrobin	1	2		1		1		1		6
Flutriafol	5									5
Cyproconazole	1	2	1							4
Difenoconazole	2	1	1							4
Fluazinam		1			2					3
Cyazofamid					2					2
Fluquinconazole	2									2
Imazalil	1		1							2
Lambda-cyhalothrin							2			2
Metalaxyl	1		1							2
Thiabendazole	1		1							2
Trifloxystrobin		1	1							2
beta-cyfluthrin	1									1
Carbetamide			1							1
Cyantraniliprole							1			1
Fludioxonil		1								1
Fluxapyroxad	1									1
Imidacloprid	1									1
Mepiquat	1									1
Propyzamide			1							1
Pyraclostrobin	1									1
Tefluthrin							1			1
Thiophanate-methyl		1								1

^a^Denominated Phoma stem canker in articles from the UK

Altogether, 29 different active substances were reported in the articles, of which three insecticides (lambda-cyhalothrin, cyantraniliprole and tefluthrin) were aimed at the treatment of insects transmitting TuYV. The active substances have changed over the years, and a few studies, particularly those concerning Sclerotinia stem rot, were excluded since the substances tested are no longer allowed for use in the EU. Tebuconazole was the most frequent active substance used and was included in 15 articles, followed by boscalid and prochloraz, which were each included in 13 articles. Azoxystrobin, metconazole and prothioconazole were included in 12, 11 and 10 articles, respectively (Table 7). For black leg, 19 different active substances were studied, and for light leaf spot, 14 and 13 substances were studied to control Sclerotinia stem rot.

The list with the different biological control agents (BCAs) tested and included in the systematic map is displayed in Table 8. In total, 17 different BCAs were used in the articles included in the systematic map, with *Bacillus subtilis* as the most common BCA tested (n = 9), mainly for Sclerotinia stem rot control (n = 5). In addition, Sclerotinia stem rot was the most studied disease, with most studies investigating the effectiveness of a range of BCAs. Black leg and Verticillium wilt were subject to five different BCAs.

**Table 8:** Number of articles included in the systematic map for biological control agents (BCAs) and targeted diseases

Biological control agent	Stem rot	Black leg^a^	Verticillium wilt	Damping-off	Black spot	Clubroot	Downy mildew	Light leaf spot	Total
*Bacillus subtilis*	5		1	1		1		1	9
Azotobacter		1		1	1		1		4
*Coniothyrium minitans*	4								4
*Trichoderma asperellum*		1		1	1		1		4
*Paecilomyces lilacinus*	2								2
*Pseudomonas fluorescens*	1		1						2
*Serratia plymuthica*		1	1						2
*Trichoderma harzianum*	1	1							2
*Trichoderma* sp.	2								2
*Bacillus cereus*	1								1
*Bacillus megaterium*	1								1
*Gliocladium catenulatum*						1			1
*Leptosphaeria biglobosa*		1							1
*Paenibacillus polymyxa*			1						1
*Stenotrophomas maltophilia*			1						1
*Talaromyces falvus*	1								1
*Trichoderma viride*	1								1

^a^Phoma stem canker

Crop rotation as an intervention was included in eight articles for managing black leg, in three articles for managing stem rot, in two articles for managing black spot, clubroot and Verticillium wilt and in one article for managing grey mould and light leaf spot (Table 6).

Among the agricultural intervention management practices, stubble management was evaluated in eight articles for black leg and in one article for Sclerotina stem rot, black spot, damping-off and Verticillium wilt (Table 6), as these diseases are caused by pathogens spread from inoculum produced on the stubble or in the soil. Sowing time was investigated as an intervention method in 11 articles, most of which concerned black leg and damping-off. Adding soil amendments was evaluated, particularly for clubroot control, with four articles concerning amendments of boron, calcium cyanamide, wood ash and calcium carbonate.

### Limitations of the map

The systematic map protocol on which this study is based was developed to capture all eligible studies on disease management in the six most important field crops in Sweden i.e., wheat, barley, oats, rapeseed, potatoes and sugar beets [26]. During the development of the protocol, different search strings were tested, and the string that was the most suitable for the selected crops was defined. No set of papers for the comprehensiveness test was compiled, and thus, this step in the ROSES procedure was not performed.

The climatic selection criteria confined the geographical distribution of the included studies to regions where the climate corresponds to Dfc, Dfb, Cfb and the future predicted Cfa climate in Sweden based on the Köppen-Geiger climatic classification zones [27]. Hence, studies performed in India, which is one of the largest OSR-producing countries in the world (Fig. 1), were excluded from the systematic map. The production of OSR in India, however, is dominated by the oilseed mustard *Brassica juncea* [41], and thus, most of the studies would not be eligible for this map.

The selection criteria for crops excluded spring OTR (*Brassica rapa*) and winter OTR [42]. During the first 20 years of the study period (1978–1998), spring OTR greatly contributed to the production of rapeseed in the Nordic countries. In 2001, OTR contributed 23%, and spring OTR contributed 30% of total Swedish oilseed acreage [7]. However, ten years later, in 2011, the acreage of spring OTR dropped to 3% of the total acreage, and the production of spring oilseed crops was dominated by OSR grown on 38% of the total acreage. The proportion of rapeseed crops has further changed, and the last 5-year averages (2015–2019) were as follows: winter OSR 94%, winter OTR < 1%, spring OSR 6% and spring OTR 1%.

Field experiments where pesticide efficiency and other agricultural cropping methods are evaluated are often published in databases that are made available for public use on special webpages, which may be difficult to identify from outside the country or region in which these studies are published. In addition, the information is often published in national languages, and thus, it was not available to us. The eligibility criteria were developed to include only studies based on pesticides allowed for use in the EU and in Sweden. Since the list of allowed active substances is constantly updated, several studies performed with older pesticides were excluded, as the substances evaluated not are allowed for use. As a result, several studies of diseases such as Sclerotinia stem rot were excluded. The search string did not specifically include the words “pesticid*”, “fungicid*” and “herbicid*”. After receiving a suggestion made by the reviewers, an updated version of the search string including these words was tested. The search captured one additional eligible article [43], which could have been included in the systematic map. Therefore, future maps should include these words in the search string.

The aim of the design of the search strategy was to capture a range of eligible studies reporting results that can directly be incorporated into plant protection strategies communicated to farmers. However, based on the criterion “articles based on field trials”, studies reporting on the development of tools and methodologies enabling rapid detection of disease outbreaks and decision support systems (DSS) were excluded. These tools are highly important for meeting the challenges posed by sustainable disease management [44] but are rarely presented together with compiled data on the yield response of different management interventions.

Breeding programmes for host resistance are one key method for controlling plant diseases [45]. Studies on breeding and pre-breeding are not included, as the advancements in these articles are indirectly implemented in crop production since new cultivars must go through registration before marketing. Registrations of new cultivars (n = 27) are included in “books, reviews and reports” (Additional file 4).

## Conclusions

Research on crop protection is often a needs-driven process, where stakeholders identify a problem or question that needs to be addressed through a systematic approach. With the growing concern over worldwide food shortages and climate change, protecting food crops against pathogens that cause epidemic diseases is of great importance [39]. This systematic map collates and catalogues existing evidence for the management of OSR in temperate climates. It provides a base of evidence useful for plant protection researchers to identify research topics in need of further studies or new topics that are not well covered by scientific studies. This systematic map also highlights diseases that are likely to increase in Sweden with climate change, such as the virus diseases TuYV and TYMV. For example, most winter OSR cultivars grown in Sweden are of European origin with the same genetic heritage, including resistance genes against plant pathogens. We can thus expect that the diseases occurring elsewhere in Europe also will occur in Sweden.

We provide a database with literature from which disease management strategies concerning OSR can be extracted and used to update plant protection recommendations. The systematic map concept offers a great potential in providing a broad overview of available crop protection methods that can be applied when developing sustainable crop protection strategies and national and regional research strategies. The maps also support the implementation of IPM and will facilitate meeting key challenges in plant pathology [45]. The strategic map concept was recently provided for oats [30] and can be used for other crops and management strategies. In the map reported here, we have focused on oilseed rape, the second most important source of vegetable oil globally [13], and on biotic stresses caused by diseases in this crop. The systematic map will support initiatives in handling the fact that production in Europe has stagnated, and it will help to show all possibilities for combating OSR diseases.

### Implications for research

The map highlights the distribution of relevant field experiments, where black leg is by far the most studied disease. The map also identifies knowledge gaps regarding diseases and management methods on which few studies have been published. Only three articles were included concerning the virus diseases TuYV and TYMV transmitted by aphids; these diseases are expected to become a more severe disease problem as the winter climate becomes milder [38]. OSR seed is routinely treated with fungicides before seeding to prevent seedlings from injuries caused by damping-off diseases when emerging. However, only six articles concerning seed treatment were included, and this is an area where more research is needed. In addition, insect pests play an important role in OSR cropping worldwide [13, 46] and are threatening the profitability of OSR, and can be subject to a complementary systematic map.

Cultivar resistance and fungicide application were the intervention methods currently most used to control OSR diseases worldwide in the studies included in the systematic map. Fungicides are effective in controlling diseases, but continuous use can lead to fungicide resistance and negative environmental impact. An increasing number of pesticides are being banned, and the substances approved for use in the EU must be effective and contribute to an increase in the yield and quality of the harvested product. For farmers, controlling diseases with fungicides incurs a cost. Crop protection strategies based on disease support as disease diagnosis and prognosis for disease development and to make the optimal decisions is increasingly important and may also have positive benefits for the environment. For some diseases, such as clubroot, no pesticide interventions are available, and quantitative disease resistance is used in winter OSR and canola. This resistance is under pressure, particularly when rotations are narrow [12], i.e., 1–2 years between OSR crops. For Sclerotinia stem rot, no resistant cultivars are available; thus, interventions are directed towards strategies based on fungicide use. One area suggested for further research is a combination of approaches with different management strategies where the use of disease support systems (DSSs) is highly valued to adapt the optimal solutions for individual decisions. A large body of results has been conducted in field trials performed by private companies or published in local languages, resulting in limited access to knowledge for the international community, and a shift towards open access publication would be valuable. Since the results are judged as being commercially sensitive, it is unlikely that these data will be available in the future. The use of a scientifically relevant study design and statistical analysis is of great value, and studies assembled from other countries or regions that address relevant crop protection measures might need to be adopted for local conditions.

### Implications for policy and management

Research on plant pathology is often based on the availability of funding in competition with other research topics related to crop production, and it is linked to the economic importance of the proposed outcome of the project. This systematic map will be an excellent tool for decision-makers and funding bodies to identify the relevance of new research topics related to plant protection that need intensified research and to optimize the allocation of available resources.

The map provides an overview of the available disease protection management interventions for OSR production in Sweden and for other regions within the climatic zones defined, and we present a new important tool for field-based advisors to provide scientifically based plant protection strategies for farmers. Collaboration between farmers, advisors and researchers is crucial for knowledge transfer, for developing relevant research questions and for establishing multi-actor platforms and participatory approaches.

## Supplementary Information

**Additional file 1: **ROSES for systematic map reports version 1.0.

**Additional file 2: **List of relevant organizational webpages and search results.

**Additional file 3: **List of pesticide active substances and biological control organisms approved for use in the EU.

**Additional file 4: **List of articles removed by tile/abstract and full text and list of articles included in the folder “books, reviews and reports”.

**Additional file 5: **Oilseed rape systematic map database.

## Data Availability

All data generated or analysed during this study are included in this published article and in its additional files.
